# Sebaceous carcinoma of the parotid gland: a case report and review of the literature

**DOI:** 10.1186/s13256-016-0946-z

**Published:** 2016-06-13

**Authors:** El Amin Marnouche, Abdelhak Maghous, Selma Kadiri, Soufiane Berhili, Asmae Touil, Fouad Kettani, Sanaa Majjaoui, Hanane Elkacemi, Tayeb Kebdani, Noureddine Benjaafar

**Affiliations:** 1 Department of Radiotherapy, National Institute of Oncology, Rabat, Morocco; 2 United Nations Center of Cytopathology, Rabat, Morocco

**Keywords:** Sebaceous carcinoma, Parotid gland

## Abstract

**Background:**

Sebaceous carcinoma is a rare malignancy primarily with aggressive growth affecting the cutaneous tissues of the periocular region. Sebaceous carcinoma of the parotid gland is exceedingly rare, with only 32 cases reported in the literature. Our case brings this total to 33.

**Case presentation:**

We present a case of a 57-year-old Moroccan woman with a firm, painless, slowly enlarging swelling at her left parotid area, with normal overlying skin and no palpable neck nodes. Parotidectomy with facial nerve preservation was performed, and microscopic examination showed sebaceous carcinoma. Then, she underwent adjuvant radiotherapy. With a follow up of 20 months, head and neck computed tomography revealed no recurrence.

**Conclusions:**

The optimal treatment is unclear. With more cases reported, clinicopathological characteristics and histogenesis are increasingly understood. Therefore the treatment for this rare tumor continues to evolve.

## Background

Sebaceous carcinoma (SC) is a rare neoplasm with aggressive growth. In 75 % of all cases [1] this tumor presents in the cutaneous tissues of the periocular region, typically on the eyelid, but it has also been described less commonly in other locations (70 % in the head and neck region [1]), including as a primary tumor of the parotid gland [2]. The parotid gland is the second most frequent site for SC in the head and neck region [3].

In 1953, Foote and Frazell first reported in a review of salivary gland tumors a case of sebaceous adenoma, specifying that the listing of a single example of this tumor type is more an anticipation, than a proof, of its actual existence [4]. Six years later, Rauch and Masshoff described a malignant counterpart (SC) in the parotid gland [5]. Up to February 2015, there have been 32 documented cases of SC in the parotid gland [3, 6–28] with our case bringing the total to 33. Its clinicopathological characteristics and histogenesis are not fully understood because of its rarity.

We present a case of SC of the parotid gland with a brief review of the literature.

## Case presentation

A 57-year-old Moroccan woman with no tobacco smoking history presented to an otolaryngology clinic with the chief complaint being a lump over the left side of her parotid region, for 9 months’ duration. Her anamnesis did not reveal a similar case in her family. A physical examination demonstrated a firm, painless, slowly enlarging swelling, with normal overlying skin and no palpable neck nodes.

**Fig. 1:**
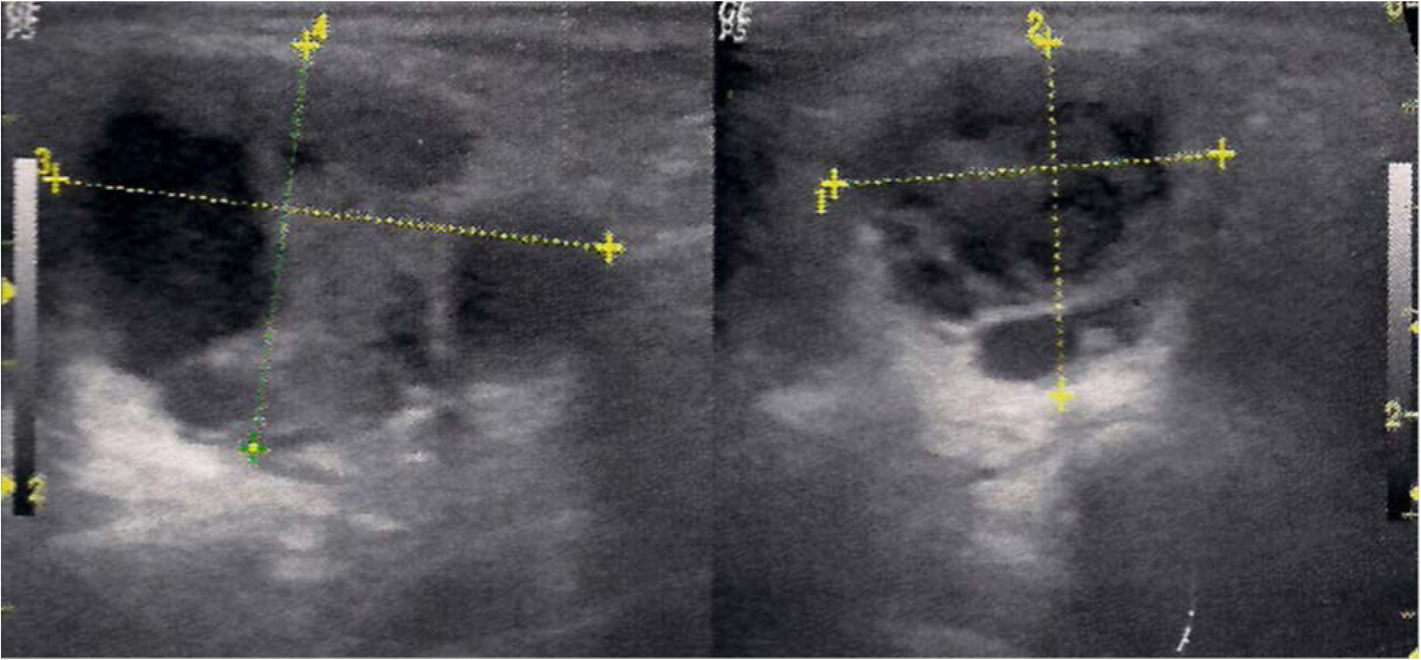
Ultrasonography revealed a mixed component nodule of the left parotid

Ultrasonography of her neck revealed a 2.7 × 2.2 cm, mixed component nodule with increased vascularity (Fig. 1). Based on clinical and ultrasonography findings, parotidectomy with facial nerve preservation was performed without lymph node neck dissection.

**Fig. 2:**
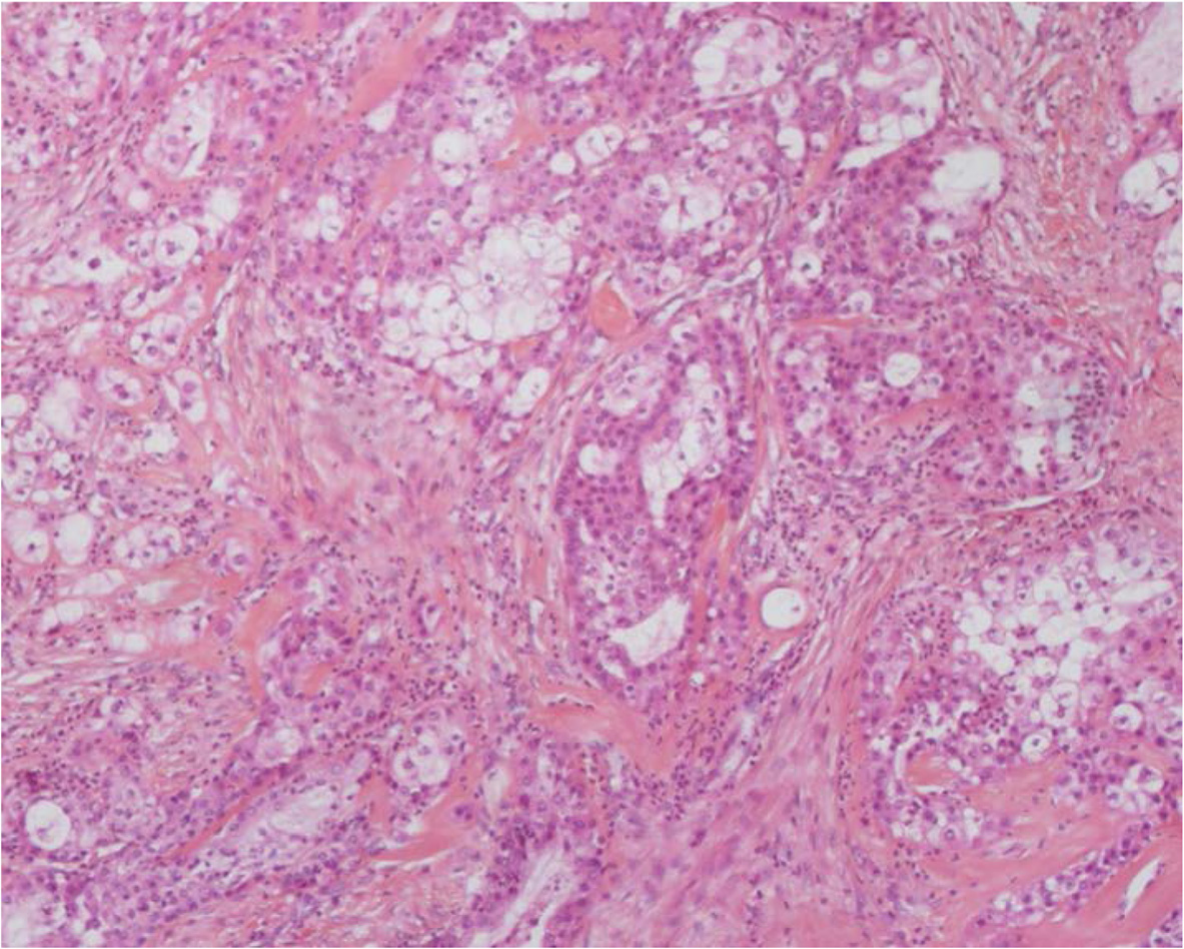
Malignant proliferation composed of cells organized in nests and bays of different sizes, with moderate to marked cytonuclear atypia, and a mixture of well-differentiated sebocytes and atypical basaloid cells

Final pathology revealed a SC that was a low-grade tumor. On macroscopic examination, the mass was ill defined and white; it measured 1.5 × 2 cm. Microscopic examination demonstrated malignant proliferation composed of cells organized in nests and bays, with moderate to marked cytonuclear atypia, and a mixture of well-differentiated sebocytes and atypical basaloid cells. Mitotic figures were present (two to four per high-power field). No vascular invasion was demonstrated (Fig. 2). Immunohistochemistry was not performed.

**Fig. 3:**
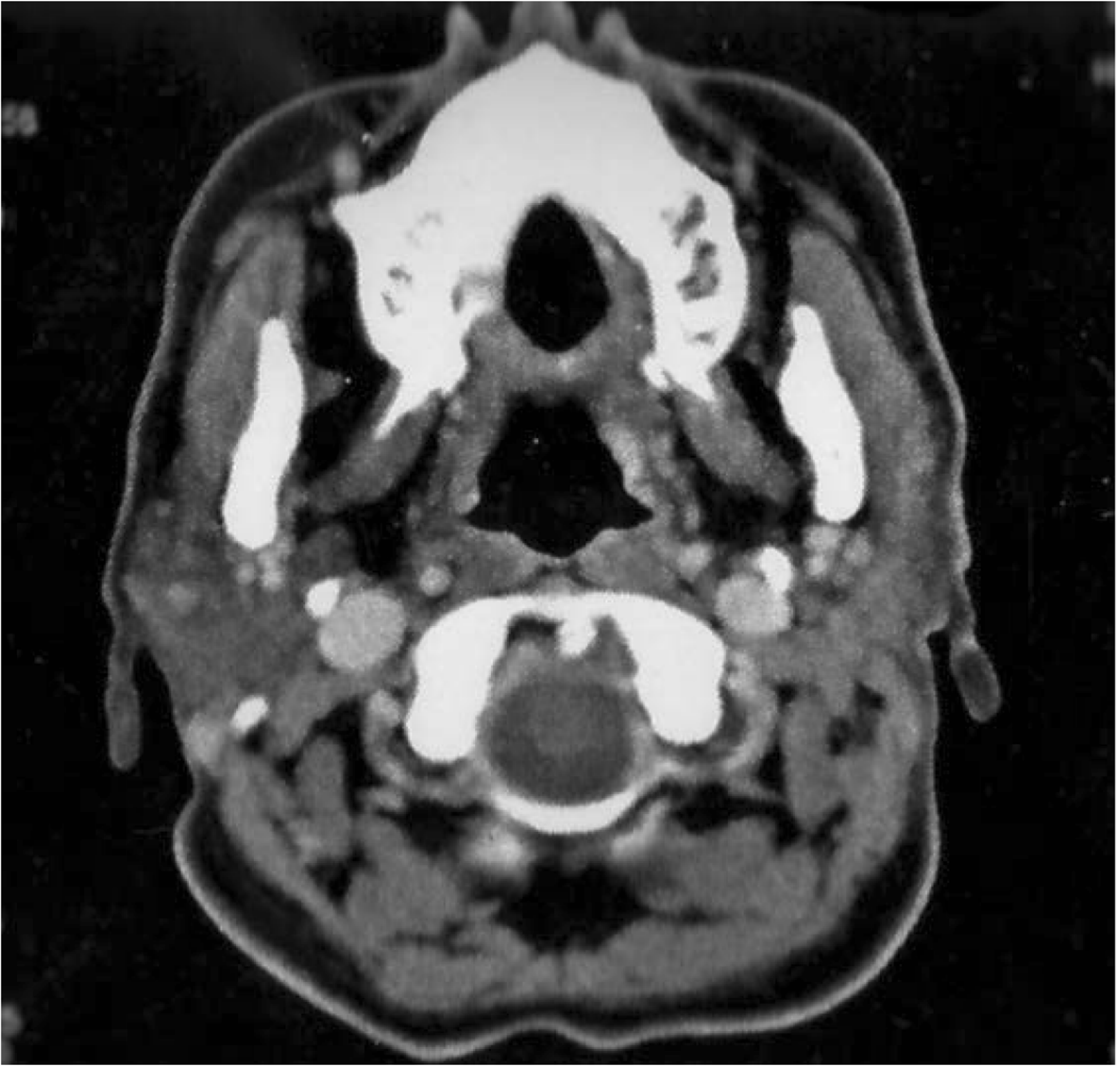
Postoperative computed tomography scan showing no residual tissue in parotid space

She was referred to our institution. We proposed a lymph node dissection but she refused further surgery. Postoperative computed tomography (CT) was performed and confirmed no residual disease (Fig. 3) and no neck nodes. A chest X-ray was also performed. Therefore, she was classified stage II according to the seventh edition of the American Joint Committee on Cancer’s (AJCC) *AJCC Cancer Staging Manual*. She completed a course of three-dimensional conformal radiation therapy, indicated by the close margins, without chemotherapy. Two oblique fields (6 MV) were used to deliver 66 Gy with 2 Gy per fraction in parotid bed and an anterior field (6 MV) to deliver 50Gy, 2Gy per a day to ipsilateral neck lymph node (level II to IV).

With a follow up of 20 months, head and neck CT revealed no recurrence and no adverse event was noted.

## Discussion

Sebaceous glands are found in an estimated 10 to 40 % of normal parotid glands and 6 to 10 % of submandibular glands [29, 30]. However, malignant sebaceous salivary gland tumors are extremely rare, making up less than 0.2 % of all major salivary glands tumors [30]. In addition to parotid gland, salivary gland SC has been identified in the submandibular gland, oral cavity, sublingual gland, vallecula, epiglottis, and hypopharynx, totaling 47 SCs of salivary origin neoplasms [6].

These sebaceous elements appeared to originate from the blind-ending intercalated and striated ducts. There is also immunohistochemical evidence to show that SC originates from pluripotential duct cells; ultrastructural and immunohistochemical observations of the tumor revealed coexistence of sebaceous and glandular differentiations in some tumor cells. Tumor cells with lipid granules often participated in the formation of glandular structures or exhibited intracytoplasmic lumina, and immunohistochemical localization of lactoferrin and secretory component, the functional markers of ductal epithelium of salivary gland, was demonstrated not only in duct-forming tumor cells but also in many sebaceous tumor cells. It seems likely that SC originates from pluripotential duct cells which can differentiate into sebaceous, ductal, and mucous cells [24].

The age range of the affected patients was found to be 17 to 93 years, the maximum incidence for all the primary salivary gland sebaceous tumors occurred in the sixth and seventh decades [20]. The sex ratio is 1:1 [11].

The patients often presented with a painless, slow-growing, asymptomatic swelling, leading to delayed diagnosis or misdiagnosis. But some had experienced pain and there were a few cases with facial paralyses [11].

On histological examination, SC ranged from 0.6 to 9.5 cm in greatest dimension and varied from yellow, tan-white, grayish-white, white, to pale pink. The tumors are frequently well circumscribed or partially encapsulated, with pushing or locally infiltrating margins. Cellular pleomorphism and cytologic atypia are uniformly present and are much more prevalent than in sebaceous adenomas. Tumor cells may be arranged in multiple variably sized nests or in sheets and have hyperchromatic nuclei surrounded by abundant clear vacuolated to eosinophilic cytoplasm. Cellular pleomorphism and atypia varies from mild to severe. Areas of cellular necrosis and fibrosis are commonly found. Perineural invasion was observed in more than 20 % of tumors, whereas vascular invasion was extremely infrequent. Rare oncocytes and foreign body giant cells with histiocytes may be observed, but lymphoid tissue with follicles or subcapsular sinuses is not seen.

Identification of sebocytes is the key morphologic clue to this entity, as is the absence of matrix protein typically seen in basal cell adenomas and pleomorphic adenomas. Neither the squamous component nor the goblet cells of mucoepidermoid carcinoma are seen in SC, and a mucin stain will be negative. SC does not show the cribriform growth pattern seen in adenoid cystic carcinoma, and is less likely to show perineural invasion. Other uncommon tumors of the parotid with sebaceous differentiation include sebaceous adenoma, which is usually sharply circumscribed, sebaceous lymphadenoma, which contains abundant lymphocytes, similar to a Warthin tumor, and sebaceous lymphadenocarcinoma, which shows adjacent areas of typical sebaceous lymphadenoma [29].

Because of its rarity, a conclusion regarding optimal therapy for this malignancy is lacking. However, treatment is based on surgery with wide surgical excision for low-grade and low-stage carcinomas. Adjunctive radiation therapy is recommended for higher stage and higher grade tumors but also in the case of positive margins. Radical parotidectomy and elective neck dissection should be considered for tumors with marked cytologic atypia or involvement of the facial nerve [6].

SCs may recur and they will rarely metastasize; at least six cases of SC of the salivary glands have been described with local recurrence and metastasis [31]. The overall 5-year survival rate is 62 %, significantly less than the survival rate for similar tumors arising in the skin and orbit (84.5 %) [32].

## Conclusions

With more cases reported, clinicopathological characteristics and histogenesis are increasingly understood. Therefore the treatment for this rare tumor continues to evolve.

## Abbreviations

CT, computed tomography; SC, sebaceous carcinoma

## References

[1] Altemani A, Vargas PA, Cardinali I, et al. Sebaceous carcinoma of the parotid gland in children: An immunohistochemical and ploidy study. Int J Oral Maxillofac Surg. 2008;37:433–40. 10.1016/j.ijom.2008.02.007 18396014

[2] Kressin MK, Coogan AC. Sebaceous carcinoma of the parotid gland. Diagn Cytopathol. 2013;41(9):803–5. 10.1002/dc.22892 22807439

[3] Mighell AJ, Stassen LF, Soames JV. Sebaceous carcinoma of the parotid gland. Dentomaxillofac Radiol. 1996;25(1):51–3. 10.1259/dmfr.25.1.9084287 9084287

[4] Foote Jr FW, Frazell EL. Tumors of the major salivary glands. Cancer. 1953;6(6):1065–133. 10.1002/1097-0142(195311)6:6<1065::aid-cncr2820060602>3.0.co;2-0 13106826

[5] Rauch S, Masshoff W. Sialoma resembling sebaceous gland [in German]. Frankf Z Pathol. 1959;69:513–25. 13739789

[6] Manteghi A, Zwillenberg S, Arquello-Guerra V. Sebaceous carcinoma of the parotid gland: A case report and review of the literature. Ear, Nose Throat J. 2014;93(6):E29–32. 24932826

[7] MacFarlane JK, Viloria JB, Palmer JD. Sebaceous cell carcinoma of the parotid gland. Am J Surg. 1975;130(4):449–501. 10.1016/0002-9610(75)90494-8 170840

[8] Tsukada Y, Delapava S, Pickren JW. Sebaceous-cell carcinoma arising in mixed tumor of parotid salivary gland. Report of a case. Oral Surg Oral Med Oral Pathol. 1964;18:517–22. 10.1016/0030-4220(64)90401-3 14198091

[9] Silver H, Goldstein MA. Sebaceous cell carcinoma of the parotid region. A review of the literature and a case report. Cancer. 1966;19(12):1773–9. 10.1002/1097-0142(196612)19:12<1773::aid-cncr2820191203>3.0.co;2-q 5927936

[10] Cheek R, Pitcock JA. Sebaceous lesions of the parotid. Report of two cases. Arch Pathol. 1966;82(2):147–50. 4287784

[11] Constant E, Leahy MS. Sebaceous cell carcinoma. Plast Reconstr Surg. 1968;41(5):433–7. 10.1097/00006534-196805000-00003 5652634

[12] Mathis H. Contribution to the knowledge about sialomas. A sebaceous carcinoma of the parotid gland [in German]. Dtsch Zahn Mund Kieferheilkd Zentralbl Gesamte. 1968;50(9):405–8. 5252697

[13] Pageaut G, Oppermann A, Carbillet JP. “Sebaceous” metaplasia of the normal, inflammatory and tumorous parotid gland [in French]. Arch Anat Pathol (Paris). 1969;17(2):101–5. 5345306

[14] Kleinsasser O, Hübner G, Klein HJ. Sebaceous cell carcinoma of the parotid gland [in German]. Arch Klin Exp Ohren Nasen Kehlkopfheilkd. 1970;197(1):59–71. 5514115

[15] Batsakis JG, Littler ER, Leahy MS. Sebaceous cell lesions of the head and neck. Arch Otolaryngol. 1972;95(2):151–7. 10.1001/archotol.1972.00770080239012 5060062

[16] Shulman J, Waisman J, Morledge D. Sebaceous carcinoma of the parotid gland. Arch Otolaryngol. 1973;98(6):417–21. 10.1001/archotol.1973.00780020431015 4757275

[17] Akhtar M, Gosalbez TG, Brody H. Primary sebaceous carcinoma of the parotid gland. Arch Pathol. 1973;96(3):161–3. 4124691

[18] Schmid KO, Albrich W. The significance of sebaceous cells and sebaceous glands in parotid tumors [author’s transl.]. Virchows Arch A Pathol Pathol Anat. 1973;359(3):239–53. 4357011

[19] Zechner G, Albegger KW. Proceedings: Sebaceous gland carcinoma of the parotid gland [in German]. Arch Klin Exp Ohren Nasen Kehlkopfheilkd. 1973;205(2):119–21. 4785475

[20] Gnepp DR, Brannon R. Sebaceous neoplasms of salivary gland origin. Report of 21 cases. Cancer. 1984;53(10):2155–70. 10.1002/1097-0142(19840515)53:10<2155::aid-cncr2820531026>3.0.co;2-f 6704903

[21] Hayashi Y, Takemoto T, Tokuoka S, et al. Sebaceous carcinoma of the parotid gland: Report of a case. Patol Clin Med. 1985;3:1135–41.

[22] Grieve TP, Saragas E, Mannell A. Sebaceous carcinoma of the parotid gland in a black patient. A case report. S Afr Med J. 1986;69(2):141–2. 3941951

[23] Granstrom G, Aldenborg F, Jeppsson PH. Sebaceous carcinoma of the parotid gland: Report of a case and review of the literature. J Oral Maxillofac Surg. 1987;45(8):731–3. 10.1016/0278-2391(87)90322-3 3475447

[24] Takata T, Ogawa I, Nikai H. Sebaceous carcinoma of the parotid gland: An immunohistochemical and ultrastructural study. Virchows Archiv A Pathol Anat Histopathol. 1989;414(5):459–64. 10.1007/BF00718631 2499103

[25] Ameline E, Amanou L, Arkwright S, et al. Sebaceous carcinoma of the parotid gland [in French]. Rev Laryngol Otol Rhinol (Bord). 1999;120(5):337–9. 10769569

[26] Siriwardena BS, Tilakaratne WM, Rajapakshe RM. A case of sebaceous carcinoma of the parotid gland. J Oral Pathol Med. 2003;32(2):121–3. 10.1034/j.1600-0714.2003.00037.x 12542836

[27] Cohn ML, Callender DL, El-Naggar AK. Sebaceous carcinoma expleomorphic adenoma: A rare phenotypic occurrence. Ann Diagn Pathol. 2004;8(4):224–6. 10.1053/j.anndiagpath.2004.06.001 15290674

[28] Takada Y. Sebaceous carcinoma of the parotid gland: a case report. Case Rep Oncol. 2015;8:106–12. 10.1159/000379742 PMC4361912 25848361

[29] Batsakis JG, el Naggar AK. Sebaceous lesions of salivary glands and oral cavity. Ann Otol Rhinol Laryngol. 1990;99:416–8. 10.1177/000348949009900517 2337322

[30] Ellis GL, Auclair P. Tumors of salivary glands. In: Rosai J, Sobin L, editors. Atlas of tumor pathology. Washington: Armed Forces Institute of Pathology; 1996. p. 130–6.

[31] Gnepp DR. My journey into the world of salivary gland sebaceous neoplasms. Head Neck Pathol. 2012;6(1):101–10. 10.1007/s12105-012-0343-x PMC3311956 22430772

[32] Boniuk M, Zimmerman LE. Sebaceous carcinoma of the eyelid, eyebrow, caruncle and orbit. Int Ophthalmol Clin. 1972;12(1):225–57. 4644889

